# IL-36α Regulates Tubulointerstitial Inflammation in the Mouse Kidney

**DOI:** 10.3389/fimmu.2017.01346

**Published:** 2017-10-23

**Authors:** Osamu Ichii, Junpei Kimura, Tadashi Okamura, Taro Horino, Teppei Nakamura, Hayato Sasaki, Yaser Hosny Ali Elewa, Yasuhiro Kon

**Affiliations:** ^1^Laboratory of Anatomy, Faculty of Veterinary Medicine, Department of Basic Veterinary Sciences, Hokkaido University, Sapporo, Japan; ^2^Department of Laboratory Animal Medicine, National Center for Global Health and Medicine, Tokyo, Japan; ^3^Department of Infectious Diseases, National Center for Global Health and Medicine, Tokyo, Japan; ^4^Department of Endocrinology, Metabolism and Nephrology, Kochi Medical School, Kochi University, Nankoku, Japan; ^5^Section of Biological Safety Research, Chitose Laboratory, Japan Food Research Laboratories, Chitose, Japan; ^6^Laboratory of Laboratory Animal Science and Medicine, School of Veterinary Medicine, Kitasato University, Towada, Japan; ^7^Faculty of Veterinary Medicine, Department of Histology and Cytology, Zagazig University, Zagazig, Egypt

**Keywords:** inflammation, distal tubule, IL-36α, tubulointerstitial lesion, unilateral ureter obstruction, folic acid

## Abstract

IL-36α, a member of the IL-1 family, is a crucial mediator of inflammatory responses. We previously found that IL-36α was overexpressed in injured distal tubules (DTs); however, its pathological function remains unclear. Herein, unilateral ureter obstruction (UUO) or folic acid (FA) injection was performed in mouse kidneys to assess the role of IL-36α in kidney injury. IL-36α mRNA and protein expression significantly increased in the kidneys within 24 h after UUO. IL-36α localized to dilated DTs. IL-36α expression significantly correlated with the progression of tubulointerstitial cell infiltration and tubular epithelium cell death in UUO kidneys and with renal dysfunction in FA-induced acute kidney injury mice. At 24 h after UUO, IL-36α^+^ DT epithelial cells showed loose intercellular digitations. IL-1RL2, an IL-36α receptor protein, localized to podocytes, proximal tubules, and DTs in the healthy kidney. IL-1RL2 was expressed in interstitial cells and platelets or extended primary cilia of DT epithelial cells in UUO kidneys. IL-36α stimulation promoted the production of IL-6 and Prss35, an inflammatory cytokine and collagen remodeling-associated enzyme, respectively, in cultured NIH3T3 fibroblasts. UUO-treated IL-36α-knockout (KO) mice showed milder kidney injury features than wild-type (WT) mice did. In UUO kidneys from IL-36α-KO mice, the expression of genes associated with inflammatory response and sensory perception was significantly different from that in WT mice. Altogether, our data indicate an association between intrarenal IL-36α overexpression and the progression of tubulointerstitial inflammations and morpho-functional alterations of DT epithelial cells. IL-36α may be a novel kidney injury marker useful for evaluating DT damages.

## Introduction

Renal histopathology is divided into glomerular or tubulointerstitial lesions (TILs). Impairment of renal function correlates with the latter, which is considered a common pathway leading to renal fibrosis (1). TILs include several morphological changes of kidney tissues such as tubular dilations and cell death as well as interstitial cell infiltration. The features of TILs differ among diseases. Drug-induced acute kidney injury (AKI), caused by antibiotics or anticancer drugs, is characterized by severe injuries of proximal tubules (PTs) (2).

Although renal function is usually evaluated by analyzing serum levels of blood urea nitrogen (BUN) and creatinine (Cr) and urinalysis, these renal function biomarkers are insufficient to detect early onset kidney diseases (3). Recent studies identified molecular markers of early renal dysfunction, inflammation, or cell damages (4, 5). Some markers were detected in whole urine and/or urinary exosomes, suggesting their potential as fluid biomarkers. Hepatitis A virus cellular receptor 1 (Havcr1, also known as Kim-1/Tim-1) and lipocalin 2 (Lcn2, also known as Ngal) are excellent markers to predict TILs in both AKI and chronic kidney disease (CKD) (4, 5).

Almost all biomarkers for TILs, including Kim-1 and Ngal, are overexpressed in PTs (4, 5). PTs are the longest segments in mammalian kidneys, and proliferation of parenchymal progenitor cells is mainly observed in the PTs among nephrons (6, 7). These data reflect the regenerative activity and capacity of PTs in response to renal injuries. In contrast, distal tubules (DTs) are relatively short segments, but crucial regulation centers for blood pressure and electrolytes. However, DT injury has not been fully examined nor distinguished from PT damages because excellent molecular markers for DT damages are lacking. Therefore, the proposed injury processes of renal tubules are based on those of PTs.

We recently demonstrated the upregulation of IL-1 family cytokines in the kidney of mouse CKD model (8–10). IL-1 family members are crucial mediators of inflammatory responses initiated from the rapid production of IL-1 family cytokines (11); therefore, these cytokines might have a potential as early mediators and markers for AKI. In fact, after surgery such as cardiopulmonary bypass, AKI was detected at 48–72 h due to serum Cr elevation. In contrast, urine level of IL-18, an intrarenal macrophage-derived IL-1 family cytokine, increased over 25-fold at 12 h and remained elevated up to 48 h after surgery (12). These data emphasize the advantages of IL-1 family members as markers of kidney diseases because of their rapid biological response to tissue injuries. IL-1 family member 6 (IL-1F6, also known as IL-36α) is overexpressed in injured DTs from various mouse kidney diseases, including lupus nephritis, diabetic nephropathy, and traumatic kidney injury (8–10). Therefore, IL-36α could be a useful marker of DT injury; however, its pathological function remains unclear.

In this study, we examined the pathological significance of IL-36α during the progression of TIL using a unilateral ureter obstruction (UUO) model, folic acid (FA)-induced AKI model, and IL-36α-knockout (KO) mice as well as *in vitro* analysis. We show that intrarenal IL-36α overexpression is associated with renal dysfunction and the progression of TILs, including morpho-functional alteration of DT epithelial cells.

## Materials and Methods

### Ethics Statement

Animal experimentation was approved by the Institutional Animal Care and Use Committee of the Graduate School of Veterinary Medicine, Hokkaido University (approval No. 16-0124). The investigators adhered to the Guide for the Care and Use of Laboratory Animals of Hokkaido University, Graduate School of Veterinary Medicine (approved by the Association for the Assessment and Accreditation of Laboratory Animal Care International).

### Genome Editing

Two single guide RNA (sgRNA) designed for the target sequences (5′-CCTAGGGTCAATCTGCAGAT-3′ and 5′-AGGGGGGATC CCACGTACAT-3′), and Cas9 mRNA were prepared as described previously (13). Briefly, an sgRNA expression vector with a T7 promoter was synthesized for the target sequence and transcribed *in vitro* using a MEGAshortscript kit (Life Technologies, Carlsbad, CA, USA). *hCas9* mRNA was synthesized using mMESSAGE mMACHINE T7 kit (Life Technologies) and was polyadenylated with polyA tailing kit (Life Technologies). The purified *hCas9* mRNAs at 100 ng/mL and each sgRNAs at 50 ng/mL targeting exon 2 and exon 3 for *Ilf6* (coding gene of IL-36α) were co-injected into the cytoplasm of the pronuclear stage eggs from C57BL/6N (B6; Japan SLC; Shizuoka, Japan), and the eggs were transferred into the oviducts of pseudopregnant ICR female mice. The founders were genotyped with primer pairs spanning the CRISPR/Cas9 cleaved site (Table S1 in Supplementary Material). The mutation of *Il1f6* gene was confirmed by sequencing analysis. To minimize the risk of off-target effects, the resulting founder (13) mouse was backcrossed to B6 mice for two generations, and the heterozygous IL-36α-KO mice were then intercrossed to produce the homozygous IL-36α-KO mice and wild-type (WT) mice that were used for this study. The genotype of the obtained littermates was confirmed by genotyping PCR analysis using specific primers for *Il1f6* mutation (Table S1 in Supplementary Material). The potential off-target site was predicted by CRISPR Design Tool (14) and listed in Table S2 in Supplementary Material.

### UUO and FA-Induced AKI Models

B6 mice were used in all *in vivo* study. UUO operation was performed to male mice at 10 weeks of age or IL-36α-KO mice at 4 weeks of ages. At 0–11 days after UUO, both kidneys and serum were collected. BrdU (100 mg/kg, i.p.) was injected to some mice 2 h before sampling. A part of each kidney was fixed in paraformaldehyde (PFA) or glutaraldehyde (GTA), and the remaining parts were stored at −80°C.

For the FA-induced AKI model, FA (800 mg/kg, i.p., Sigma-Aldrich; St. Louis, MO, USA) was injected to male B6 mice at 10 weeks of age. At 8 h after injection, the serum, urine, and kidneys were collected. A part of each kidney was fixed in 4% PFA, and the remaining parts were stored at −80°C. Urinary Cr was determined in FA models by the Creatinine Companion (Exocell; Philadelphia, PA, USA). Soluble proteins were extracted by using RIPA lysis buffer (Santa Cruz; Dallas, TX, USA) from urinary sediments and urinary exosomes. Urine (400 µL) was centrifuged at 21,000 × *g* for 10 min, and its sediments and supernatants were used. Lithium dodecyl sulfate-sample buffer and sample reducing reagent (Thermo Fisher Scientific; Waltham, MA, USA) were added to the samples or directly added to urinary supernatant. The serum levels of BUN and Cr were measured with Fuji Drichem (Fujifilm Medical Co. Ltd.; Tokyo, Japan).

### Cell Culture

M-1, MES13, NIH3T3, and AI cell lines [kindly provided from Dr. J.B. Kopp, National Institutes of Health (NIH)] were maintained as previously described (15, 16). Immortalized mouse PT cells (Figure S1 in Supplementary Material) and 209/MDCT (American Type Culture Collection; Manassas, VA, USA) cells were maintained in REGM BulletKit (Lonza; Basel, Switzerland) and DMEM/F12 medium containing 10% fetal bovine serum (FBS) and 1× penicillin/streptomycin (PS; Thermo Fisher Scientific), respectively. For 209/MDCT, after stimulation by NaCl, KCl, H_2_O_2_, or LPS (Wako; Osaka, Japan) under FBS-free condition, cells were collected for RNA and protein analysis, or were fixed by using 4% PFA and stained by immunofluorescence method using IL-36α antibody (Table S3 in Supplementary Material), phalloidin (Life Technologies), and Hoechst 33342 (Dojindo; Kumamoto, Japan). NIH3T3 cells were stimulated by recombinant mouse IL-36α (7059-ML-010, R&D Systems; Minneapolis, MN, USA), and the culture medium and cells were collected for RNA and protein analysis. IL-6 in the culture medium was measured by ELISA (IBL; Gunma, Japan).

### Microarray

Total RNA was extracted from frozen UUO and collateral control (Cont) kidneys of B6 mice at 7 days or WT and IL-36α-KO mice at 48 h. One pooled sample collected from four kidneys was analyzed in each group. Gene expression was analyzed using a GeneChip Mouse Gene 2.0 ST Array (Affymetrix; Santa Clara, CA, USA). Microarray signals were normalized by using the RMA algorithm. Generic gene ontology (GO) term finder (http://go.princeton.edu/cgi-bin/GOTermFinder) was used for GO analysis.

### Real-time PCR

Total RNA from frozen kidneys was isolated and used as a template to synthesize cDNA using ReverTra Ace qPCR RT Master Mix (Toyobo; Osaka, Japan). Quantitative PCR analysis was performed by using Brilliant III SYBR Master Mixes for kidneys (Agilent; Santa Clara, CA, USA) or TaqMan Real-Time PCR Master Mixes for cells (Thermo Fisher Scientific) and specific primers or probes (Table S1 in Supplementary Material) with an MX3000P system (Agilent). The specificity of each PCR reaction was confirmed by melting curve analysis. The expression data were normalized to the expression levels of *Actb or Gapdh*.

### Immunoblotting

From collected kidneys, cells, and urines, soluble proteins were extracted by using RIPA lysis buffer (Santa Cruz). Lithium dodecyl sulfate-sample buffer and sample reducing reagent (Thermo Fisher Scientific) were added to the samples. Immunoblotting was performed by using the NuPAGE electrophoresis system (Thermo Fisher Scientific) with the antibodies listed in Table S3 in Supplementary Material. Immunocomplexes were detected by using Typhoon Variable-Mode Imagers (GE Healthcare; Little Chalfont, UK). The intensity of band was quantified using Image J (NIH).

### Histopathology

Paraffin-embedded kidney sections were stained with periodic acid Schiff (PAS) or Masson’s trichrome for histopathological analysis, and the area of renal pelvis (RP) was measured using ImageJ (NIH) or BZ-H3M (Keyence; Osaka, Japan). Sections were analyzed by immunohistochemistry or immunofluorescence using primary antibodies listed in Table S3 in Supplementary Material as described previously (9, 16) and evaluated for the number of B220^+^ cells, Gr1^+^ cells, Iba1^+^ cells, single strand DNA (ssDNA)^+^ cells, Caspase 3^+^ cells, and IL-36α^+^ tubules. The signals detected in areas positive for CD3, BrdU, alpha-smooth muscle actin (αSMA), and IL-36α were measured by using ImageJ (NIH) or BZ-H3M (Keyence).

### Electron Microscopy

Four percent PFA/0.1 M phosphate buffer (PB) containing 2.5% GTA or 4% PFA/0.1 M containing 0.5% GTA was used for the fixation of transmission electron microscopy (TEM) and immunoelectron microscopy (IEM) specimens, respectively. For IEM, kidneys were fixed for 4 h, and PFA was replaced with 0.1 M PB containing 10–30% sucrose. The kidneys were incubated overnight, and then embedded by using O.C.T. Compound (Sakura Finetech; Tokyo, Japan). Immunohistochemistry using 5-μm thick cryosections and streptavidin-biotinylated horseradish peroxidase complex (Nichirei; Tokyo, Japan) was performed to detect IL-36α. TEM specimens and kidney sections for IEM were post-fixed with 1% OsO_4_ and embedded in Quetol 812 (Nisshin EM; Tokyo, Japan), and the ultrathin sections were stained with or without uranyl acetate and lead citrate for TEM and IEM, respectively.

For scanning electron microscopy (SEM), specimens were fixed with 2.5% GTA/0.1 M PB and cut into 200-µm sections by vibratome, and then post-fixed with OsO_4_ and dehydrated in critical point dyer (HCP-2; Hitachi, Tokyo, Japan). For IEM using SEM, specimens were fixed with 0.5% PFA and 0.25% GTA. Immunohistochemistry was performed to detect IL-36α using 5-μm thick paraffin sections, IL-36α primary antibody, biotinylated secondary antibody, gold conjugated streptavidin-biotin, and gold enhance kit (Nanoprobe; Yaphank, NY, USA). After post-fixation by 0.5% OsO_4_ and dehydration, ion-spattered sections were observed under SEM.

### Statistical Analysis

The results are displayed as the mean ± SE and were statistically analyzed by using a Mann–Whitney *U* test (*P* < 0.05). The correlation between two parameters was analyzed by using Spearman’s rank correlation test (*P* < 0.05).

## Results

### IL-36α Is Produced by the Epithelium of DTs in UUO Kidneys

**Figure 1:**
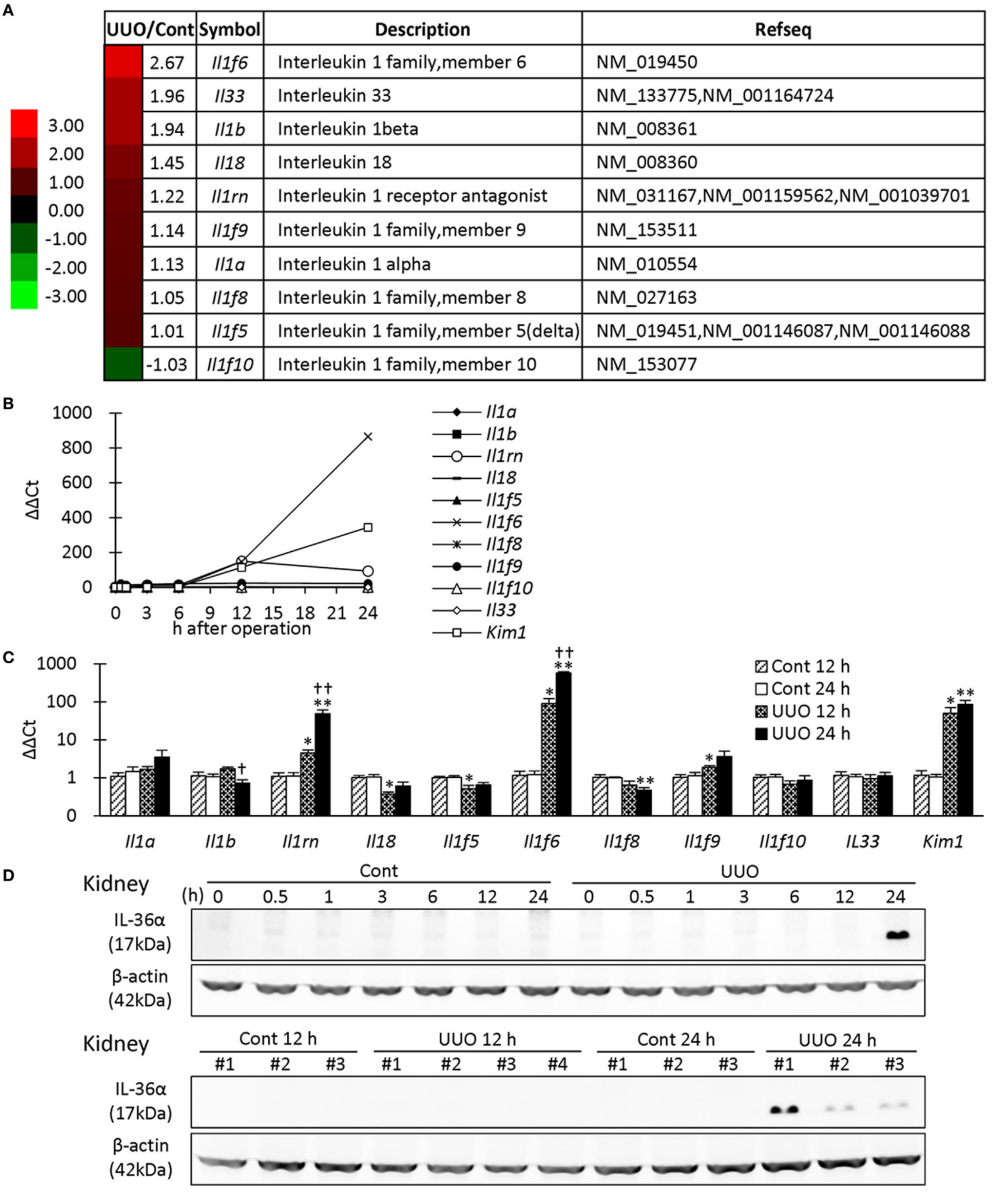
Expression of IL-1 family members in unilateral ureter obstruction (UUO) kidneys. **(A)** Gene expression of IL-1 family members analyzed by microarray at day 7 after UUO. The values indicate the fold increase of UUO kidney to Cont kidney. Heatmap scale bars indicate the relative changes. **(B)** Time course of mRNA expression of IL-1 family members and Kim-1 in UUO kidneys (*n* = 1). **(C)** Relative mRNA expression of IL-1 family members and *Kim-1* in Cont and UUO kidneys. Values = mean ± SE. A significant difference from the Cont is indicated by *(*P* < 0.05) or **(*P* < 0.01). A significant difference from 12 h in the same group is indicated by ^†^(*P* < 0.05) or ^††^(*P* < 0.01). *q* ≥ 4 kidneys. **(D)** Immunoblotting analysis of IL-36α and β-actin expression in Cont and UUO kidneys.

First, microarray analysis was performed in mouse kidneys at 7 days after UUO. Among the IL-1 family, *Il1f6* was the most upregulated gene in UUO kidneys; 2.67-fold vs. Cont kidneys (Figure 1A). Similar to *Kim-1, Il1f*6 mRNA expression in the kidneys increased from 12 h after UUO, and its elevation was prominent at 24 h compared to that of other members (Figure 1B). At 12 h, *Il1rn, Il1f6, Il1f9*, and *Kim-1* mRNA expression was significantly higher in UUO kidneys than that in Control kidneys, but that of *Il18* and *Il1f5* was decreased (Figure 1C). At 24 h, *Il1rn, Il1f6*, and *Kim-1* mRNA expression was more significantly increased in UUO kidneys. IL-36α was detected in the kidneys from 24 h after UUO (Figure 1D).

**Figure 2:**
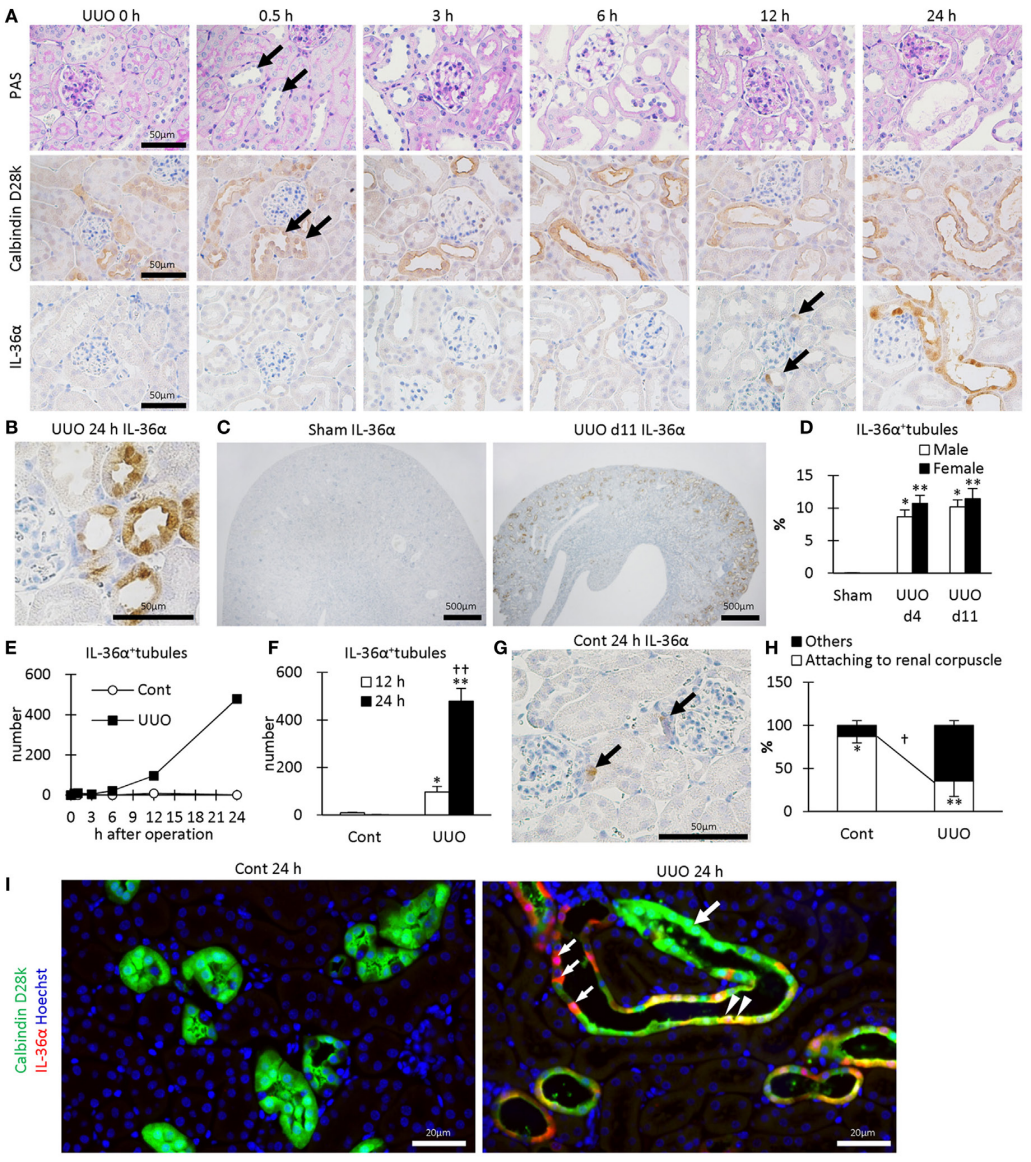
Protein expression and localization of IL-36α in unilateral ureter obstruction (UUO) kidneys. **(A)** Renal histopathology after UUO. In periodic acid Schiff staining, dilated distal tubules (DTs) are observed from 0.5 h after UUO (arrows). Positive immunostaining for calbindin D28k, a DT marker, is observed in DTs, and almost all of them were dilated from 0.5 h after UUO (arrows). IL-36α^+^ immunostaining is observed in DTs, especially in the macula densa (MD), from 12 h after UUO (arrows). This immunostaining spreads to the entire DTs at 24 h after UUO. **(B)** Immunohistochemistry for IL-36α in the UUO kidney at 24 h after UUO. IL-36α^+^ immunostaining is observed in the cytoplasm, and some nuclei are also stained. **(C)** Immunohistochemistry for IL-36α in the UUO kidney at day 11 after UUO. IL-36α^+^ tubules are numerous in UUO kidneys compared to Cont kidneys. **(D)** The number of IL-36α^+^ tubules in Cont and UUO kidneys. Values = mean ± SE. A significant difference from the Cont in same sexes is indicated by *(*P* < 0.05) or **(*P* < 0.01). No sex-related difference is detected. *n* = 5 (kidneys). **(E)** Time course of IL-36α^+^ tubule number in Cont and UUO kidneys. **(F)** The number of IL-36α^+^ tubules in Cont and UUO kidneys. Values = mean ± SE. A significant difference from the Cont is indicated by *(*P* < 0.05) or **(*P* < 0.01). A significant difference from 12 h in the same group is indicated by ^†^(*P* < 0.05) or ^††^(*P* < 0.01). *n* ≥ 5 kidneys. **(G)** Immunohistochemistry for IL-36α^+^ tubules in Cont kidney. In a few Cont kidneys, IL-36α^+^ immunostaining is occasionally observed in the MD at 24 h after UUO (arrows). **(H)** Probability of IL-36α^+^ tubules in DT segments attached to the renal corpuscles and the other DT segments. Values = mean ± SE. A significant difference from others is indicated by *(*P* < 0.05) or **(*P* < 0.01). A significant difference between Cont kidneys and UUO kidneys in the same group is indicated by ^†^(*P* < 0.05) or ^††^(*P* < 0.01). *n* = 5 (kidneys). **(I)** Immunofluorescence for calbindin D28k and IL-36α. No IL-36α^+^ staining is detected in Cont kidney. In UUO at 24 h, dilated calbindin D28k^+^ tubules (green, large arrow) are also positive for IL-36α (yellow, arrowheads), and some of them show decreased calbindin D28k^+^ staining (red, small arrows).

In UUO kidneys, the dilated tubules were observed at 0.5–24 h after UUO (Figure 2A) and were positive for calbindin D28k, a DT marker. IL-36α^+^ immunostaining appeared in DTs, first in the macula densa (MD), from 12 h after UUO, and the immunostaining spread to surrounding DT segments at 24 h (Figures 2A,B). In DTs, IL-36α^+^ immunostaining was observed in the cytoplasm and in some nuclei (Figure 2B). Increased IL-36α protein expression remained until 11 days after UUO without sex-related differences (Figures 2C,D). The number of IL-36α^+^ tubules increased from 12 h after UUO and was significantly higher in UUO kidneys than in Cont kidneys at 12 and 24 h (Figures 2E,F). In some Cont kidneys, a small number of cells were IL-36α^+^ in DTs, especially close to the MD (Figure 2G). The probability of IL-36α^+^ DT segments was significantly higher in DT segments attached to the renal corpuscle compared to the other DT segments in Cont kidneys; however, the latter DT segments showed higher values than the former DT segments in UUO kidneys (Figure 2H). These data indicated that DT segments attached to the renal corpuscle, including the MD, were initiation sites of IL-36α expression, which spread to other DT segments as kidney injury progressed. At 24 h after UUO, IL-36α expression was scarce in Cont kidneys, but clearly co-localized with calbindin D28k expression in UUO kidneys (Figure 2I).

### Morphological Alteration of IL-36α Expressing Epithelial Cells in UUO Kidneys

**Figure 3:**
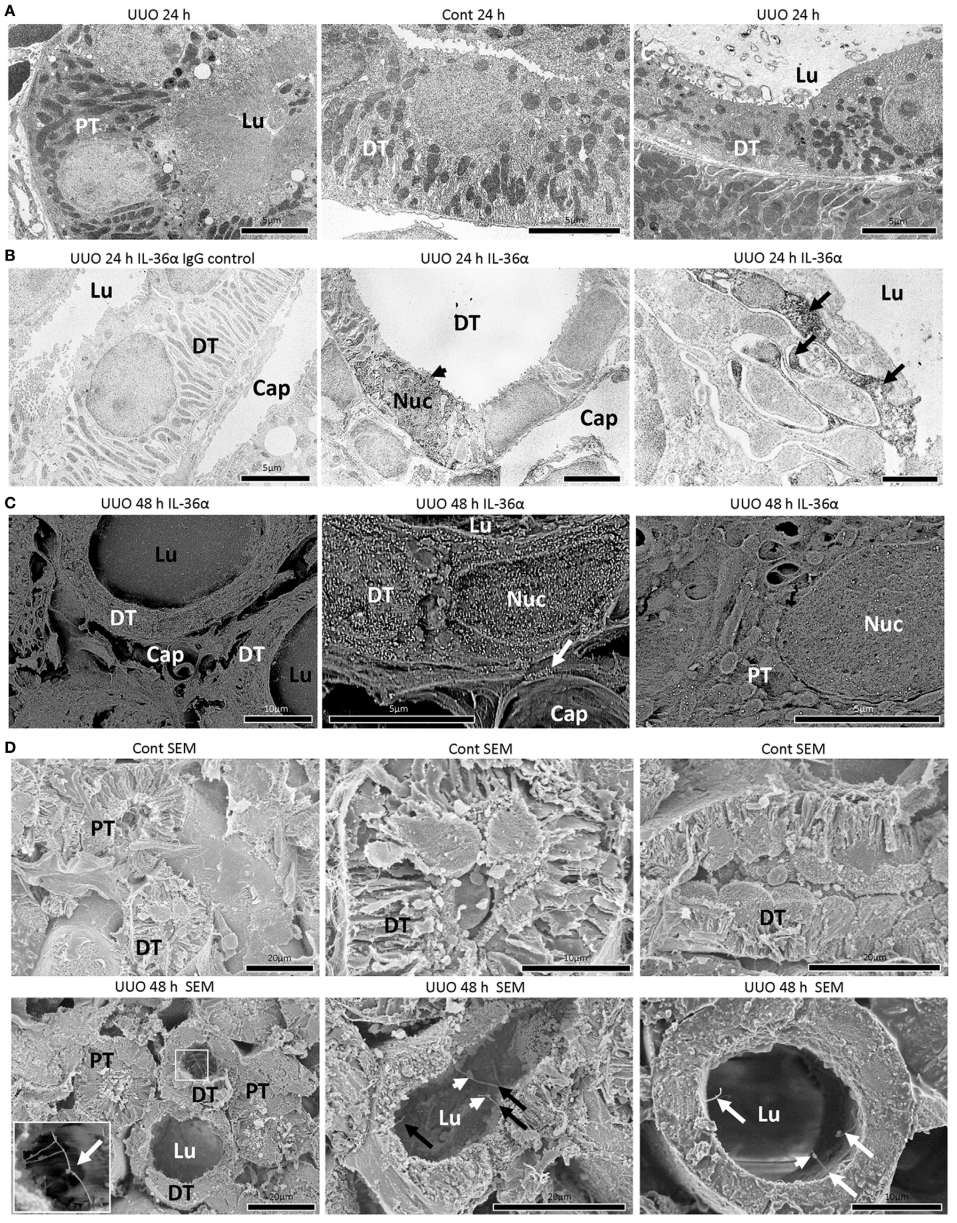
Morphological characteristics of IL-36α expressing epithelial cells in unilateral ureter obstruction (UUO) kidneys. **(A)** Transmission electron microscopy images of the kidneys at 24 h after UUO. Proximal tubule (PT) epithelial cells show normal structures in UUO kidneys. Distal tubule (DT) epithelial cells show well-developed basal infolding and regular mitochondria arrangement in Cont kidneys. In DTs of UUO kidneys, dilated tubular lumen, squamous feature, vacuolar structures, randomized mitochondria arrangement and basal infolding, and lamellar structures in the lumen are observed in epithelial cells. Lu, lumen. **(B)** Immunoelectron microscopy (IEM) images of the kidney at 24 h after UUO. No positive reaction is observed in IgG-isotype control staining. The DT epithelial cells strongly stained for IL-36α in the cytoplasm and nucleus (arrowhead) showed loose interdigitation, randomized mitochondria arrangement, and basal infolding (arrows). BM, basement membrane; Lu, lumen; Cap, peritubular capillary; Nuc, nucleus. **(C)** IEM images of the kidney at 48 h. For IEM using scanning electron microscopy (SEM), IL-36α^+^ nanogold particles are observed in dilated DTs, and these particles localize to the cytoplasm and nucleus as well as extracellular regions beside the BM (arrow) and peritubular capillary, and they are scarce in PTs. Lu, lumen; Cap, peritubular capillary; Nuc, nucleus. **(D)** Ultrastructure of Cont and UUO kidneys at 48 h under SEM. In Cont kidneys, DT epithelial cells showed well-developed basal infoldings and narrow lumens. In UUO kidneys, DT epithelial cells showed dilated lumen and long primary cilia (arrows), and some of them showed connections (insets) and bulge structure (arrowheads).

In TEM, the structures of PT epithelial cells remained normal after 24 h of UUO, but that of DT epithelial cells presented a dilated tubular lumen and squamous features (Figure 3A). IEM clarified that DT epithelial cells strongly expressing IL-36α in the cytoplasm and nucleus showed loose intercellular digitations and randomized mitochondria arrangements (Figure 3B). IEM using SEM showed that IL-36α^+^ nanogold particles were observed in dilated DTs; These particles localized to the cytoplasm and nucleus as well as in extracellular regions beside the basement membrane and peritubular capillary (Figure 3C). Furthermore, long primary cilia were observed at the apical portion of DT epithelial cells in UUO kidneys rather than in Cont kidney at 48 h by SEM, and some of these cilia presented bulges and were interconnected (Figure 3D).

### IL-36α Overexpression Correlates with Renal Dysfunction in FA-Induced AKI Kidneys

**Figure 4:**
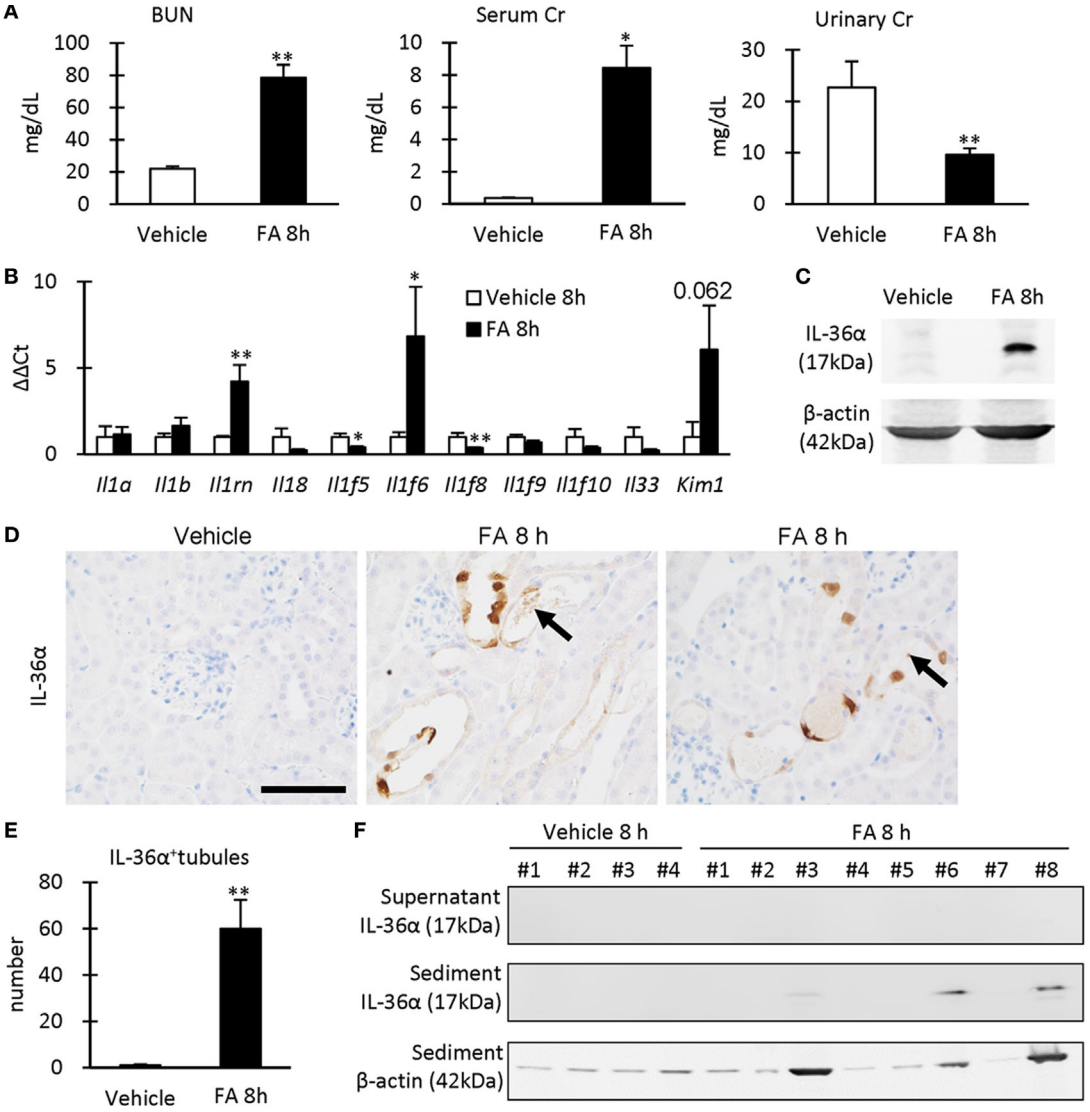
IL-36α expression and renal dysfunction in folic acid (FA)-induced AKI mice. **(A)** Serum levels of blood urea nitrogen and creatinine (Cr) and urinary level of Cr in vehicle control and FA groups after 8 h of FA injection. Values = mean ± SE. A significant difference from the vehicle control is indicated by *(*P* < 0.05) or **(*P* < 0.01) (*n* = 5–8). **(B)** Relative mRNA expression of IL-1 family members and *Kim-1* in the kidneys of vehicle control and FA groups at 8 h after FA injection. Values = mean ± SE. A significant difference from the vehicle control is indicated by *(*P* < 0.05) or **(*P* < 0.01) (*n* = 5–8). **(C)** Immunoblotting analysis of IL-36α and β-actin expression in the kidneys of vehicle control and FA groups at 8 h after FA injection. **(D)** Immunohistochemistry for IL-36α in the kidneys of vehicle control and FA groups at 8 h after FA injection. IL-36α^+^ immunostaining is observed in the cytoplasm, and some nuclei are also stained. Arrows show IL-36α^+^ cell debris. **(E)** The number of IL-36α^+^ tubules in the kidneys of vehicle control and FA groups at 8 h after FA injection. Values = mean ± SE. A significant difference from the vehicle control is indicated by **(*P* < 0.01). *n* = 5–8. **(F)** Immunoblotting analysis of IL-36α and β-actin expression in the urinary samples of vehicle control and FA groups at 8 h after FA injection.

After 8 h of FA injection, serum levels of BUN and Cr were significantly increased in the FA groups compared to that in the vehicle groups, and urinary Cr was significantly decreased in the former groups (Figure 4A). At 8 h after FA injection, *Il1rn* and *Il1f6* mRNA expression was significantly increased, but that of *Il1f5* and *Il1f8* was decreased in the kidneys of FA-AKI groups compared to that in the vehicle groups (Figure 4B). *Kim-1* mRNA expression also tended to increase after FA injection without statistical significance (*P* = 0.062). IL-36α protein expression in the kidneys was increased at 8 h after FA injection (Figures 4C–E), and IL-36α^+^ immunostaining was observed in DT epithelial cells as well as in luminal cell debris (Figure 4D). In the urine, IL-36α protein was detected in the urinary sediment of FA-AKI individuals showing increased urinary cells suggested by increased β-actin expression, but not in their supernatant (Figure 4F, see #3, 6, 8). Table 1 summarizes the correlation between IL-36α expression and renal dysfunction of the FA-AKI model. *Il1f6* mRNA expression significantly correlated with the serum levels of BUN and Cr and urinary Cr. IL-36α protein expression also significantly correlated with serum level of BUN and urinary Cr.

**Table 1:** Relationship between IL-36α expression and renal dysfunction in the folic acid (FA)-induced AKI model.

Indices		FA-induced AKI model		
		Renal function		
		Blood urea nitrogen	Serum creatinine (Cr)	Urinary Cr
*Kim-1* mRNA	ρ	0.660	0.850	−0.660
	*P*	0.018*	0.016*	0.020*
	*N*	12	7	12
*Il1f6* mRNA	ρ	0.660	0.780	−0.830
	*P*	0.020*	0.041*	0.01<**
	*N*	12	7	12
IL-36α protein	ρ	0.880	0.685	−0.710
	*P*	0.01<**	0.090	0.010*
	*N*	12	7	12

*Male B6 mice at 10 weeks of age were analyzed after FA injection for 8 h. ρ, Spearman’s rank correlation coefficient*.

*P < 0.05.

**P < 0.01.

### IL-36α Overexpression in UUO Kidneys Correlates with Interstitial Inflammation

**Figure 5:**
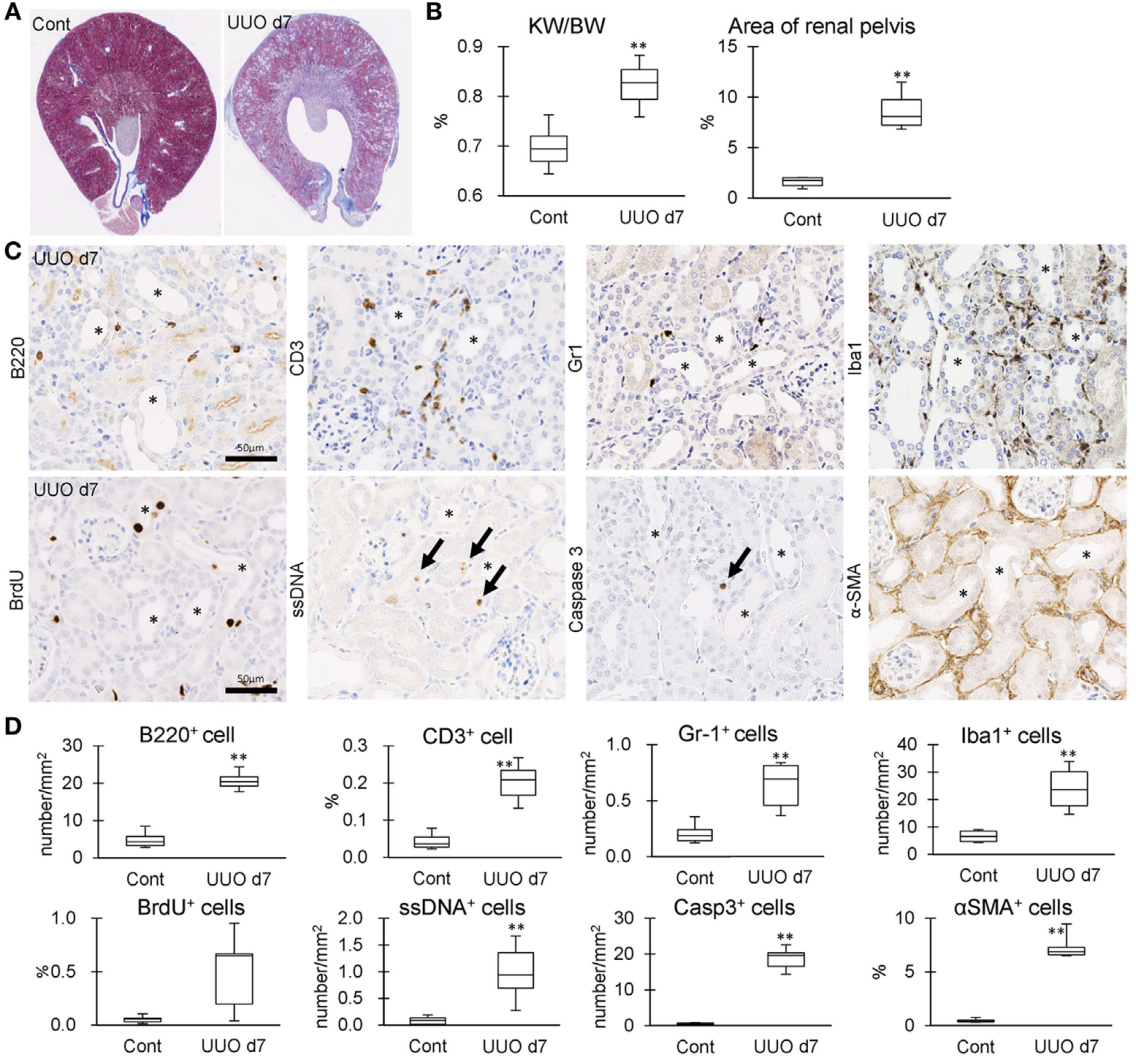
Tubulointerstitial inflammation in unilateral ureter obstruction (UUO) kidneys. **(A)** Renal histopathology at day 7 after UUO. Masson’s trichrome stain. The dilations of renal pelvis (RP) and increase of fibrotic area (blue) are prominent in UUO kidneys compared to Cont kidneys. **(B)** The ratio of KW/BW and area of RP. Values = mean ± SE. A significant difference from the Cont is indicated by **(*P* < 0.01). *n* = 6 kidneys. **(C)** Immunohistochemistry for B220, CD3, Gr1, Iba1, BrdU, single strand DNA, Caspase3, and αSMA in UUO kidneys. Arrows indicate immune-positive cells. Bars = 20 µm. **(D)** The quantified indices of immunopositive cells. Values = mean ± SE. A significant difference from the Cont is indicated by **(*P* < 0.01). *n* = 6 kidneys.

The correlation between IL-36α expression and kidney injury parameters was examined at 7 days after UUO because UUO kidneys at 7 days show severe interstitial inflammation. The ratio of kidney weight (KW)/body weight (BW) and the area of RP were significantly increased in UUO kidneys compared to those in Cont kidneys (Figures 5A,B). In UUO kidneys, infiltration of B220^+^ B-cells, CD3^+^ T-cells, Gr1^+^ granulocytes, and Iba1^+^ macrophages was observed at TILs (Figure 5C). Furthermore, BrdU^+^ proliferating cells and ssDNA^+^ or Caspase 3^+^ dead cells were detected in the tubular epithelium, and αSMA^+^ myofibroblasts increased. The quantified parameters, except for BrdU^+^ cells, and KW/BW, or the area of the RP, were significantly higher in UUO kidneys than in Cont kidneys (Figure 5D) and positively correlated with the number of IL-36α^+^ tubules (Table 2).

**Table 2:** Relationship between IL-36α^+^ tubule numbers and pathological indices in unilateral ureter obstruction (UUO) kidneys.

Indices		Weights of body and kidneys		Infiltration of immune cells				Proliferation or death of cells			Fibrosis and dilation of RP	
		BW	KW/BW	B220^+^	CD3^+^	Gr1^+^	Iba1^+^	BrdU^+^	Caspase3^+^	ssDNA^+^	αSMA^+^	RP
IL-36α^+^ tubules	ρ	0.086	0.741**	0.748**	0.860**	0.783**	0.832**	0.371	0.839**	0.792**	0.727**	0.832**
	*P*	0.872	0.006	0.005	0.001<	0.003	0.001	0.236	0.001	0.002	0.007	0.001

*Male C57BL/6N mice at 10 weeks of age were analyzed at day 7 after UUO*.

*KW, kidney weight; BW, body weight; BrdU, bromodeoxyuridine; ssDNA, single strand DNA; SMA, smooth muscle actin; RP, renal pelvis; ρ, Spearman’s rank correlation coefficient*.

**P < 0.01. N = 6. Six control and six UUO kidneys were analyzed.

### IL-36α Is Induced by LPS and Stimulates IL-6 *via* IL-1RL2

**Figure 6:**
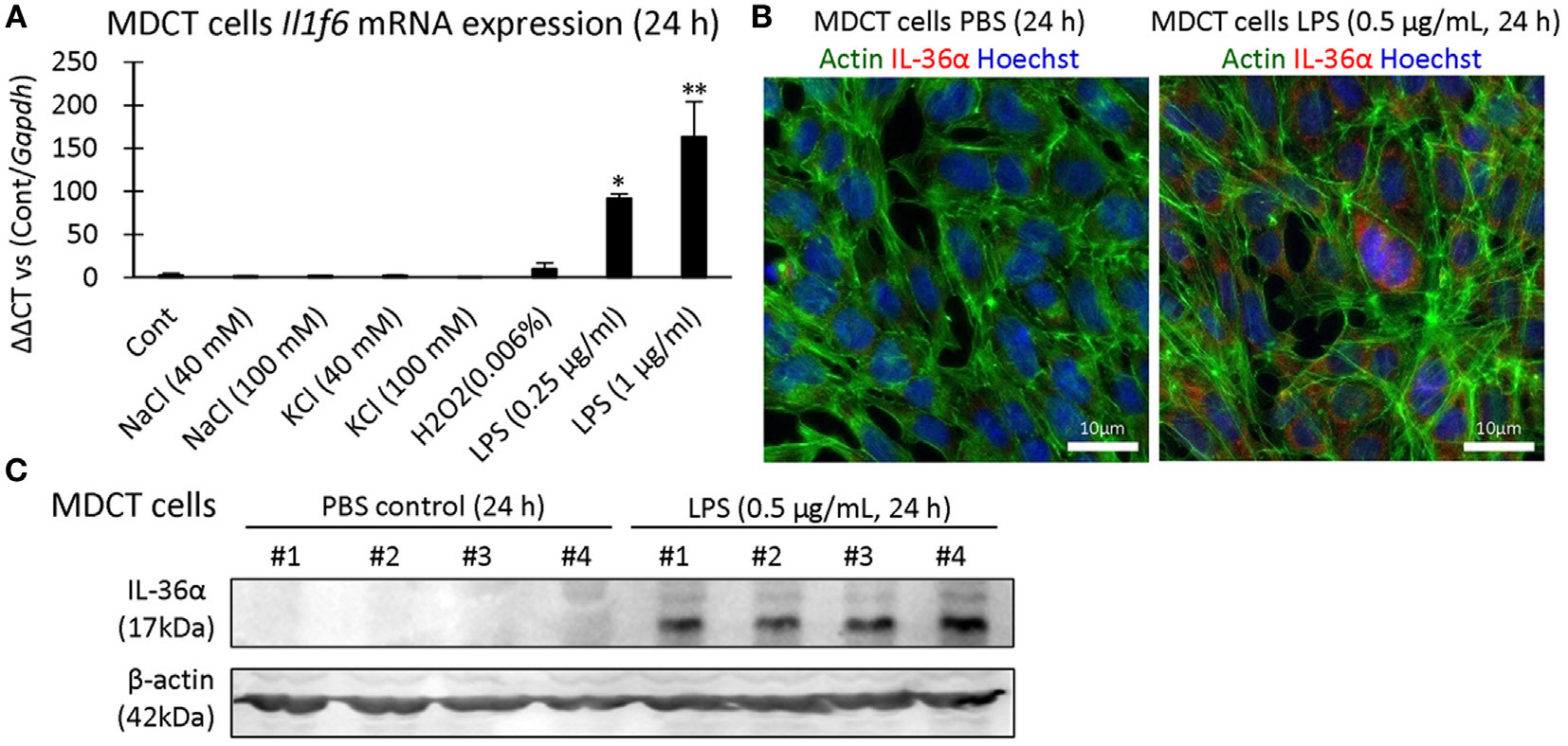
Induction of IL-36α *in vitro*. **(A)** *Il1f6* mRNA expression in 209/MDCT cells after stimulation with various agents for 24 h. A significant difference with PBS control (Cont) is indicated by *(*P* < 0.05) or **(*P* < 0.01) (*n* = 4). **(B)** Immunofluorescence in 209/MDCT cells after PBS or LPS exposure for 24 h. Green, red, and blue indicate pan-actin, IL-36α, and nuclei, respectively. **(C)** Immunoblotting for IL-36α and β-actin using cell lysates from 209/MDCT cells after PBS or LPS exposure for 24 h.

IL-36α induction was examined in 209/MDCT, a mouse DT epithelial cell line, and LPS stimulation for 24 h strongly induced IL-36α mRNA and protein (Figures 6A–C).

**Figure 7:**
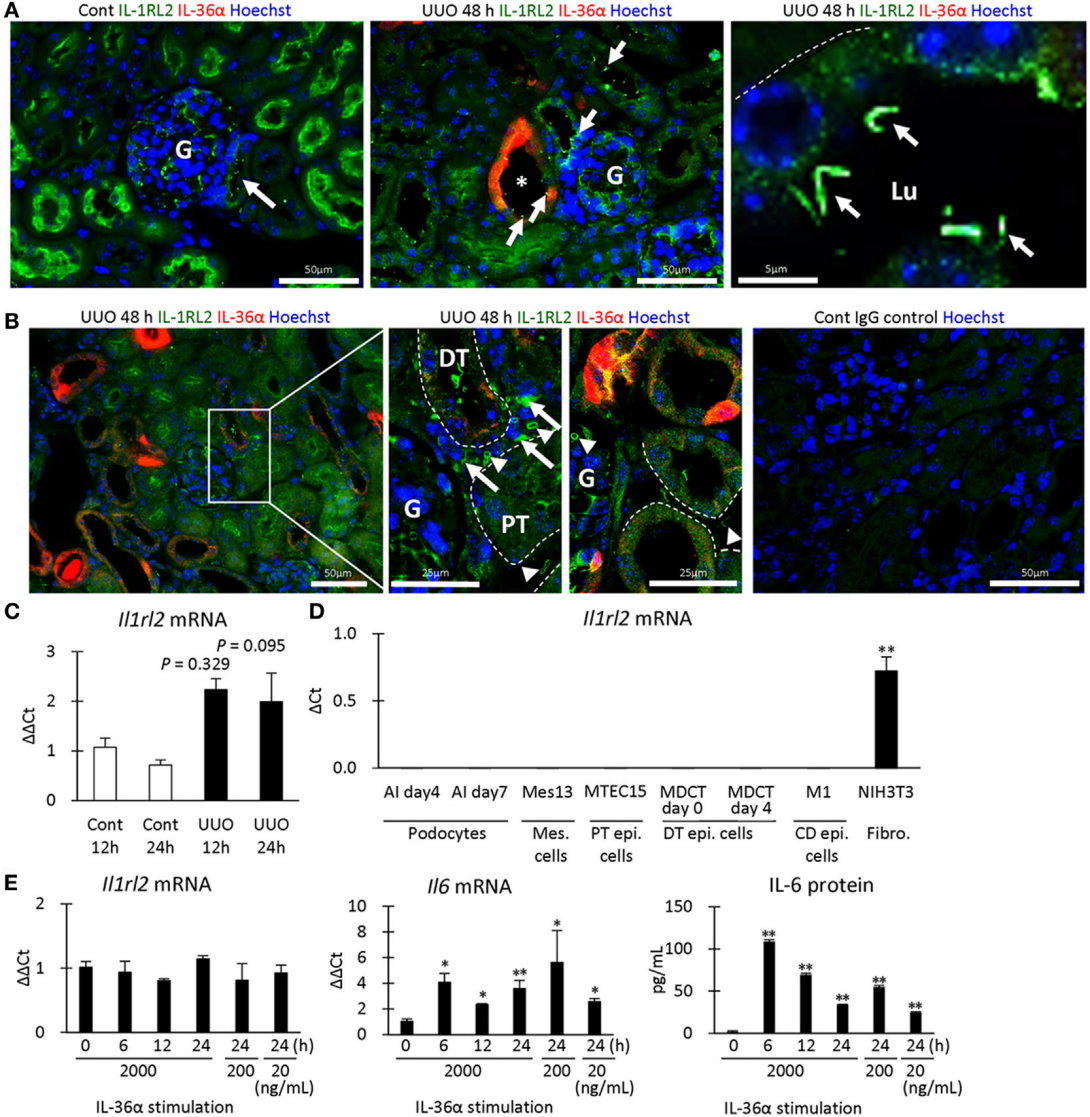
IL-1RL2 expression in the mouse kidney and induction of IL-6 production by IL-36α stimulation *in vitro*. **(A)** Immunofluorescence for IL-36α and IL-1RL2 in Cont and unilateral ureter obstruction (UUO) kidneys at 48 h. Green, red, and blue indicate IL-1RL2, IL-36α, and nuclei, respectively. Arrows indicate the dotted or linear IL-1RL2^+^ reactions. G, glomerulus; Lu, lumen. **(B)** Immunofluorescence for IL-36α and IL-1RL2 in Cont and UUO kidneys at 48 h. Green, red, and blue indicated IL-1RL2, IL-36α, and nuclei, respectively. Arrows and arrowheads indicate the IL-1RL2^+^ interstitial cells and platelets, respectively. G, glomerulus. **(C)** Relative mRNA expression of *Il1rl2* in Cont and UUO kidneys. Values = mean ± SE. *n* ≥ 4 (kidneys). **(D)** Relative mRNA expression of *Il1rl2* in mouse cell lines. Values = mean ± SE. *n* = 4. A significant difference with other cell lines is indicated by **(*P* < 0.01). Mes, mesangial; Epi, epithelial; CD, collecting duct; Fibro, fibroblast. **(E)** Relative mRNA expression of *Il1rl2* and *Il6* and medium IL-6 levels in IL-36α-exposed NIH3T3 cells. Values = mean ± SE. *n* = 4. A significant difference with 0 h is indicated by *(*P* < 0.01) or **(*P* < 0.01).

Next, the expression of IL-1RL2, a receptor of IL-36α, was examined in UUO kidneys at 48 h. IL-1RL2^+^ immunostaining was detected in PTs, podocytes, platelets, and interstitial cells (Figures 7A,B). Interestingly, dotted or rod-like IL-1RL2^+^ immunostaining was observed at the apical portion of the epithelial cells of DT, including the MD in UUO and Cont kidneys at 48 h (Figure 7A), indicating IL-1RL2 expression in primary cilia. At 12 and 24 h after UUO, *Il1rl2* mRNA expression tended to increase in UUO kidneys compared to that in Cont kidneys without statistical significance (Figure 7C). Among the examined mouse cell lines, *Il1rl2* mRNA expression was detected only in NIH3T3, a fibroblast line (Figure 7D). Therefore, IL-36α stimulation was performed in NIH3T3 cells (Figure 7E). *Il1rl2* mRNA expression was not affected, but *Il6* mRNA expression in cell lysates and IL-6 protein level in the culture medium significantly increased from 6 h after IL-36α stimulation.

### IL-36α-KO Mice Show Mild TILs in UUO Kidney

**Figure 8:**
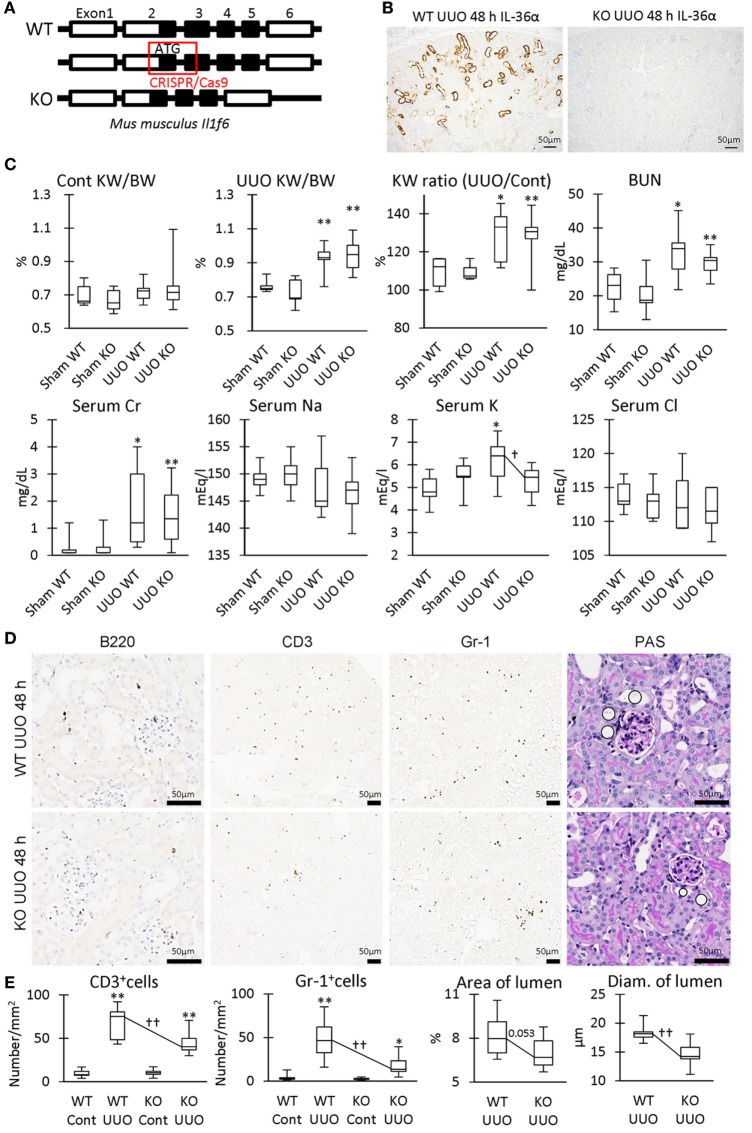
Renal pathology in unilateral ureter obstruction (UUO)-treated IL-36α- knockout (KO) mice. **(A)** Genomic structure of IL-36α. Parts of exon 2 and exon 3 are deleted by CRISPR/Cas9 system to generate IL-36α-KO mice. **(B)** Immunohistochemistry for IL-36α in the kidneys of wild-type (WT) and IL-36α-KO mice at 48 h after UUO. **(C)** Indices for renal pathology and function in Sham and UUO groups at 48 h. Median. Box = 25th and 75th percentiles. Bars = min and max values. A significant difference from the Sham group in same genotype is indicated by *(*P* < 0.05) or **(*P* < 0.01). A significant difference between WT and IL-36α-KO in UUO group is indicated by ^†^(*P* < 0.05). *n* ≥ 7 mice. The ratio of UUO kidney weight (KW) to Cont KW is expressed as KW ratio (UUO/Cont). **(D)** Renal histopathology at 48 h after UUO. Immunohistochemistry and periodic acid Schiff (PAS) stain. In PAS staining, the dilation of distal tubules (DTs) (circles) was milder in UUO kidneys compared to WT kidneys after UUO. **(E)** Indices for renal histopathology in Cont kidney and UUO kidney of WT and IL-36α-KO mice. Median. Box = 25th and 75th percentiles. Bars = min and max values. A significant difference from the Sham group in the same genotype is indicated by *(*P* < 0.05) or **(*P* < 0.01). A significant difference between WT and IL-36α-KO in UUO group is indicated by ^††^(*P* < 0.01) (*n* ≥ 7 mice).

Parts of exon 2 and exon 3 were deleted by the CRISPR/Cas9 system to generate IL-36α-KO mice (Figure 8A). At 48 h after UUO, KO mice expressed no IL-36α (Figure 8B). KW/BW, KW ratio (UUO/Cont), BUN, and serum Cr, were significantly higher at 48 h in the UUO group than in the Sham group in both WT and KO mice (Figure 8C). However, serum potassium concentration was significantly increased in the UUO group of WT mice, but not in that of KO mice compared with each Sham group, and a significant difference was observed between WT and KO mice in the UUO group. CD3^+^ T-cells and Gr1^+^ granulocytes were detected in 48 h UUO kidney in both WT and KO mice (Figure 8D), but infiltration of these cells and renal tubule dilation were significantly milder in UUO kidneys of KO mice than in WT mice (Figure 8E).

Table 3 summarized the GO analysis of genes differently expressed in the kidneys of WT and KO mice at 48 h after UUO. Genes involved in inflammatory response and acute inflammatory response were significantly downregulated (under 1.5-fold, *P* < 0.01) in the kidneys of KO mice compared to that in the kidneys of WT mice. Further, genes involved in sensory perception or detection of chemicals were significantly upregulated or downregulated (over or under 1.5-fold, *P* < 0.01). In particular, genes belonging to the olfactory receptor (Olfr) and vomeronasal receptor (Vmnr) family were frequently downregulated or upregulated (Figure S2 in Supplementary Material).

**Table 3:** Gene ontology (GO) analysis of genes showing altered expression in the kidneys of wild-type (WT) and IL-36α knockout (KO) mice after unilateral ureter obstruction (UUO) at 48 h.

Comparison	Ontology aspects	GO term	Cluster frequency (%)	Genome frequency (%)	Corrected *P*-value
Downregulated genes in UUO-treated kidney of IL-36α-KO mice	Process	Inflammatory response	8.50	2.50	0.00088
		Acute inflammatory response	3.50	0.50	0.0056
		G-protein coupled receptor signaling pathway	17.40	7.80	0.00044
		Sensory perception	15.80	7.60	0.00931
	Function	Signaling receptor activity	17.40	8.50	0.00086
		Signal transducer activity	18.90	9.70	0.00103
		Receptor activity	18.10	9.40	0.00217
		Molecular transducer activity	18.10	9.40	0.00217
		Transmembrane signaling receptor activity	16.20	8.20	0.00334
		Transmembrane receptor activity	16.20	8.30	0.00548
	Component	NA			

Two hundred and fifty nine genes were downregulated in the kidneys of IL-36α-KO mice compared with that that in the kidneys of WT mice; 24,424 genes were examined; Bonferroni correction for *P*-values. NA: not annotated					

Upregulated genes in UUO-treated kidney of IL-36α-KO mice	Process	Sensory perception of chemical stimulus	29.00	5.90	1.50E−34
		Sensory perception of smell	23.80	4.60	1.28E−28
		Sensory perception	30.00	7.60	1.96E−28
		Neurological system process	31.60	9.20	1.01E−25
		G-protein coupled receptor signaling pathway	27.40	7.80	4.29E−22
		System process	33.20	11.70	9.47E−21
		Detection of chemical stimulus involved in sensory perception	5.20	1.20	0.00132
		Detection of chemical stimulus	5.20	1.30	0.0041
		Signal transduction	34.90	23.50	0.00418
		Multicellular organismal process	44.60	32.70	0.0079
	Function	Olfactory receptor activity	23.80	4.50	5.13E−30
		Receptor activity	31.30	9.40	7.54E−25
		Molecular transducer activity	31.30	9.40	7.54E−25
		Transmembrane signaling receptor activity	29.00	8.20	8.46E−25
		Transmembrane receptor activity	29.00	8.30	3.76E−24
		Signaling receptor activity	29.00	8.50	2.26E−23
		Signal transducer activity	29.30	9.70	4.62E−20
		Odorant binding	10.70	1.90	1.16E−13
		Pheromone binding	2.60	0.40	0.00356
		Serine-type endopeptidase inhibitor activity	2.90	0.50	0.00365
		Endopeptidase inhibitor activity	3.60	0.80	0.00506
		Endopeptidase regulator activity	3.60	0.80	0.00708
		Peptidase inhibitor activity	3.60	0.80	0.00814
		G-protein coupled receptor activity	6.80	2.60	0.00961
	Component	Integral component of membrane	37.50	23.40	2.69E−06
		Intrinsic component of membrane	37.80	24.00	6.19E−06
		Membrane part	39.10	28.60	0.00736

Three hundred seven genes were upregulated in the kidneys of IL-36α-KO mice compared to that in the kidneys of WT mice; 24,424 genes were examined; Bonferroni correction for *P*-values					

### IL-1F6/IL-36a Stimulates IL-6 and Prss35 Productions *via* IL-1RL2

**Figure 9:**
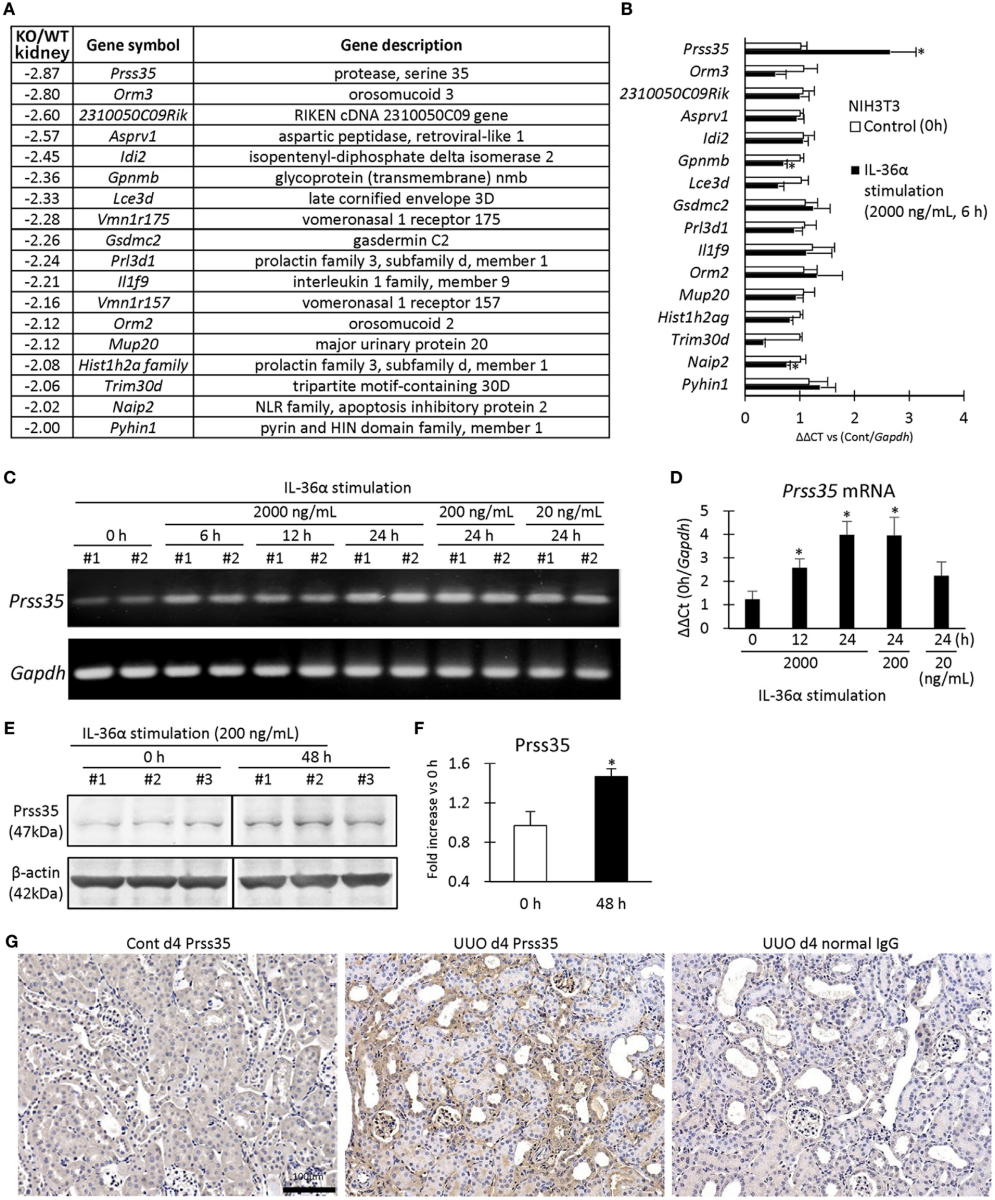
Induction of Prss35 by IL-36α stimulation *in vitro* and unilateral ureter obstruction (UUO) kidneys *in vivo*. **(A)** Genes downregulated (twofold down) in microarray in the kidneys of IL-36α- knockout (KO) mice compared with that in WT mice at 48 h after UUO. **(B)** Relative mRNA expression of genes downregulated in panel **(A)** in IL-36α-exposed NIH3T3 cells. Values = mean ± SE (*n* = 4). A significant difference with 0 h is indicated by *(*P* < 0.05). **(C)** mRNA expression of *Prss35* in IL-36α-exposed NIH3T3 cells. **(D)** Relative mRNA expression of *Prss35* in IL-36α-exposed NIH3T3 cells. Values = mean ± SE (*n* = 4). A significant difference with 0 h is indicated by *(*P* < 0.05). **(E)** Immunoblotting for Prss35 and β-actin using cell lysates from NIH3T3 cells after IL-36α exposure for 0 and 48 h. **(F)** Prss35 levels in cell lysate from IL-36α-exposed NIH3T3 cells. Values = mean ± SE (*n* = 4). A significant difference with 0 h is indicated by *(*P* < 0.05). **(G)** Immunohistochemistry for Prss35 in Cont and UUO kidneys of WT mice. Prss35^+^ staining was strong in interstitial fibroblasts in the kidneys of UUO at 4 days after operation. No positive reaction is observed in IgG-isotype control staining.

Figure 9A summarizes the genes differentially expressed in the kidneys of WT and IL-36α-KO mice at 48 h after UUO in microarray analysis. In particular, protease serine 35 (Prss35) mRNA expression was decreased in KO mice, suggesting Prss35 as a candidate downstream molecule of IL-36α signaling. NIH3T3 stimulated by IL-36α showed increased Prss35 mRNA and protein expression (Figures 9B–F), and Prss35^+^ immunostaining was detected in interstitial fibroblasts and DTs of UUO kidneys (Figure 9G).

## Discussion

In this study, IL-36α was the most upregulated gene in UUO mouse kidneys among IL-1 family members. IL-36α belongs to the IL-36 subfamily, including IL-1F8/IL-36β, IL-1F9/IL-36γ, and IL-1F5 (IL-36 receptor antagonist; IL-1F5/IL-36Ra) in humans (17, 18). Various epithelial cells express IL-36 cytokines, which activate NF-κB and MAPK pathways and promote inflammatory responses in respiratory diseases, arthritis, or dermatitis (17–20). Our results revealed that *Il1f6* and *Il1rn* mRNA expression increased, but that of *Il1f5* and *Il1f8* decreased in the mouse kidneys injured by UUO and FA. Therefore, IL-1 family members coordinately alter their transcriptional status to maintain the balance between activation and inhibition of inflammation.

The present study demonstrates the significant correlation between IL-36α expression and TIL scores, indicating that IL-36α plays a crucial role in TIL progression. Furthermore, IL-36α localized to DTs, and DT segments, including the MD, were suggested as the initiation sites of IL-36α production in the early response to kidney injury. There is no clear evidence about cytokines being expressed in the MD; however, cyclooxygenase 2, a strong inducer of cytokines *via* the arachidonic acid cascade, is constitutively expressed in the MD, indicating its potentials as an inflammatory regulator (21, 22).

Our *in vitro* study revealed that LPS, a toll-like receptor ligand, induced IL-36α production in 209/MDCT, suggesting that the activation of NF-κB or MAPK pathways (23) *via* toll-like receptor might be an upstream event of IL-36α induction. Furthermore, IL-1RL2 localized to renal epithelial cells, interstitial cells, and platelets in the mouse kidney. IL-36α increases in serum from patients with inflammatory disease such as Sjögren’s syndrome (24, 25) and in the urine of patients with TILs (26). Our IEM observation also indicated IL-36α excretion from the injured DT epithelium; therefore, these IL-1RL2-expressing cells receive IL-36α signals directly in a paracrine/autocrine manner or indirectly *via* the urine and blood. In the examined cell lines, NIH3T3 fibroblasts showed *Il1rl2* expression and produced IL-6 in response to IL-36α stimulation. Thus, we speculate that interstitial fibroblasts participate in the IL-36α-IL-1RL2/IL-6 cascade. Importantly, IL-1RL2 localization was clear at expanded cilia of DT epithelial cells after UUO. Therefore, *in vivo*, disease conditions might be important to maintain IL-1RL2 cellular expression.

IL-36α-KO mice presented milder kidney disease features than WT mice after UUO, including serum potassium levels and TILs. DTs are important segments for potassium excretion, and increased serum potassium levels were noted in both CKD and AKI (27). Furthermore, GO analysis confirmed decreased gene expression patterns associated with the inflammatory response in the IL-36α-KO kidneys. Therefore, the data obtained with KO mice indicated the attenuation of renal dysfunction and inflammation because of IL-36α deficiency from DTs. Importantly, Prss35 increased in UUO mouse kidney and fibrosis-associated fibroblasts (28). Moreover, Prss35 expression decreased in UUO kidneys of IL-36α-KO mice compared with WT mice. Further, Prss35 significantly increased after IL-36α stimulation in cultured fibroblasts, suggesting Prss35 as a novel downstream molecule of IL-36α. Prss35^+^ immunostaining was detected in the interstitial fibroblasts of UUO kidneys and was reported as a regulator of renal fibrosis *via* collagen degradation (28). Thus, IL-36α may regulate inflammation and the remodeling of tubulointerstitial structures *via* Prss35 production.

A recent study reported the amelioration of TILs *via* the NLRP3 inflammasome and IL-23/IL-17 axis in UUO kidneys of IL-1RL2-KO mice (26). This phenotypic amelioration in these receptor-KO mice was more drastic than our data using ligand-KO mice. Additionally, *Il23a* and *Il17a* gene expression in UUO kidneys was similar in WT and IL-36α-KO mice in microarray analysis (1.24-fold and 1.02-fold, compared with WT mice, respectively). IL-1RL2 can also bind to IL-1F8/IL-36β and IL-1F9/IL-36γ (18, 19). Therefore, these family members might compensate the lack of IL-36α in UUO kidney of KO mice.

Interestingly, epithelial cells of dilated DT showed expanded primary cilia, and the gene expression patterns of Olfr and Vmnr in UUO kidneys were significantly altered between WT and IL-36α-KO mice. Numerous family members of these sensory proteins were identified (over 1,000 members of Olfr in mice); these proteins localize to the cilia on the tips of olfactory sensory neurons, recognizing various ligands such as short chain fatty acids (29). Consistently, the kidney expresses Olfr family members, and some of them participate in blood pressure regulation *via* the renin–angiotensin system (29). Importantly, UUO for 8 days caused injury and dilation of DTs accompanied with a significant increase of cilial length (30, 31). IL-1RL2 was detected at the cilium of epithelial cells of dilated DTs in the UUO kidney; therefore, expanded cilia of DT cells may increase their sensitivity to urine flow and ligands, including IL-36α, to alter their morpho-function in response to injury.

IL-36α expression was positively correlated with serum levels of BUN and Cr in FA-induced AKI models. The *P* and ρ values of IL-36α expression with these parameters were comparable to those of Kim-1. Kim-1 and Ngal are PT-derived excellent makers for kidney injuries (4, 5). An increase in IL-36α was reported in the urine from patients with TILs (26). Our study showed that urinary sediments from FA-induced AKI mice contained IL-36α. Importantly, IL-36α is also strongly expressed in the epithelium of the skin, esophagus, thymus, and uterus, showing normal histology without inflammation in healthy mice (8). N-terminal processing is required for full agonist activity of IL-36α protein (32). IL-36 family members are activated by protease such as neutrophil granule-derived proteases (32, 33). Therefore, accelerated enzymatic processing may also be required to induce kidney diseases. Furthermore, IL-36α was also detected in the nuclei of injured DT epithelial cells. The nuclear translocation was also reported for IL-1α and IL-33, indicating their function in the transcriptional regulation of other cytokines (34). To solve these limitations, the accurate level of urine IL-36α should be determined by developing an ELISA system and candidate enzymes for intrarenal IL-36α processing as well as the function of nuclear IL-36α in kidney disease should be clarified.

In conclusion, IL-36α overexpression in DT is closely associated with the progression of tubulointerstitial inflammation. DTs are crucial regulation centers for blood pressure and electrolytes. The detection of DT damage focusing on IL-36α expression and combination with that of PT damages by using Kim-1 and Ngal in the kidney could lead to more accurate and earlier histopathological evaluation of kidney injuries.

## Ethics Statement

Animal experimentation was approved by the Institutional Animal Care and Use Committee of the Graduate School of Veterinary Medicine, Hokkaido University (approval No. 16-0124). The investigators adhered to the Guide for the Care and Use of Laboratory Animals of Hokkaido University, Graduate School of Veterinary Medicine (approved by the Association for the Assessment and Accreditation of Laboratory Animal Care International).

## Author Contributions

OI and HS conceived and performed experiments and analyzed data. OI, JK, TH, YE, and YK conceived experiments. TO and TN created knockout mice and analyzed data, respectively. All authors were involved in writing the paper and had final approval of the manuscript.

## Conflict of Interest Statement

The authors declare that the research was conducted in the absence of any commercial or financial relationships that could be construed as a potential conflict of interest.
