# Supplementary material for: IL-36α Regulates Tubulointerstitial Inflammation in the Mouse Kidney
Source: Front Immunol. 2017 Oct 23;8:1346. doi: 10.3389/fimmu.2017.01346 (PMC5660075; doi:10.3389/fimmu.2017.01346)

Supplemental Table 1. Primers and TaqMan probes.

Primers for *in vivo* analysis					
**Symbol**	**Gene ID**	**Forward primer (5′-3′)**	**Reverse primer (5′-3′)**	**bp**	**Application**
Actb	11461	TCCTTCGTTGCCGGTCCACA	TGGGCCTCGTCACCCACATA	196	Real-time PCR Normalization
Gapdh	14433	CCGCATCTTCTTGTGCAG	TGCCGTGAGTGGAGTCATAC	199	Real-time PCR Normalization
Il1a	16175	GCCATTGACCATCTCTCTCTG	TGCTTGACGTTGCTGATACTG	169	Real-time PCR
Il1b	16176	AGCTTCAGGCAGGCAGTATC	CCATGAGTCACAGAGGATGG	147	Real-time PCR
Il1rn	16181	CAAGCTGTGCCTGTCTTGTG	TGTTGTGCAGAGGAACCATC	184	Real-time PCR
Il1f5	54450	TCAAGGAGGAAGCCAGTGC	GTGAGGTGCAGAGGAACCAG	191	Real-time PCR
Il1f6	54448	CAGCATCACCTTCGCTTAGAC	AGTGTCCAGATATTGGCATGG	143	Real-time PCR
Il1f8	69677	TGCTTCTGCTGTGTTGAGATG	AACCAGCCAGGATAGAGGAC	164	Real-time PCR
Il1f9	215257	CCAGTCAGCGTGACTATCCTC	ATGGCTTCATTGGCTCAGG	193	Real-time PCR
Il1f10	215274	CCAACTGCAGGAATGTGCTC	CCTCGGTTAGGAAGGATACAGAC	158	Real-time PCR
Il18	16173	AGTAAGAGGACTGGCTGTGACC	AACTCCATCTTGTTGTGTCCTG	174	Real-time PCR
Il33	77125	GCTGATGGTGAACATGAGTCC	CTCCTATGTAAGTGCCAGGAAG	188	Real-time PCR
*Havcr1**	171283	ACCAATGGACATCGTGTCACC	GGGTCTTCTTGGAGGACGTG	219	Real-time PCR
Mmp9	17395	CCTGGAACTCACACGACATC	ACCTGGTTCACCTCATGGTC	121	Real-time PCR
Tnf	21926	CGAGTGACAAGCCTGTAGCC	GAGAACCTGGGAGTAGACAAGG	167	Real-time PCR
Prss35	244954	ACGGGAGGATACAGTAAGCATC	CTGTTTCCGACCCATCAGAGA	117	Real-time PCR
Orm3	18407	GAGAGAGAATGGGACCCTCTTCA	TCTCTAGCACTCTCAGGTGGA	71	Real-time PCR
Gpnmb	93695	AGCATGGAAAGTCTCTGCGG	ACATCACGAAATCGCTTGGC	86	Real-time PCR
Trim30d	209387	GAGGGAGTTGGTGAGAGACC	AGGACATAATTTACACCCTGCTTCA	90	Real-time PCR
Pyhin1/Ifi209	236312	ACTCCAAAAAGACCGTCCAGC	TTTTCAGTCTCTGTCTCTTGGCTAT	121	Real-time PCR
Lrp2	14725	TGCCCATGTGCTCCAGCACGCAGT	TGGGAAAGCGAGTCGGGCAAGCGGA	987	RT-PCR Supplemental figure 2
Ggt1	14598	TGCCTTGTGCGAGGTGTTCTGCCG C	TGGCAGCCACAGCACTGCCATCCT	623	RT-PCR Supplemental figure 2

Aqp1	11826	TCGCCCAGTGTGTGGGAGCCATCG T	TGCCTACCCCAAGGAAGGCTGCGGT	921	RT-PCR Supplemental figure 2
Gapdh	14433	CGACTTCAACAGCAACTC	GCCGTATTCATTGTCATACCAG	106	RT-PCR Supplemental figure 2
Il1f6	54448	TTGTGGATAAAAGGCTTGGAC	CTCACCCCAAGCTGTAGCAC	448	Genotyping for IL-36α-KO mouse

TaqMan probes for *in vitro* analysis

Symbol	Gene ID	Catalog number	Company	bp	Application
Actb	11461	Mm02619580_g1	Applied Biosystems (MT, USA)	63	Real-time PCR Normalization
Gapdh	14433	Mm99999915_g1	Applied Biosystems (MT, USA)	109	Real-time PCR Normalization
Il1f6	54448	Mm00457645_m1	Applied Biosystems (MT, USA)	103	Real-time PCR
Il1rl2	107527	Mm00519245_m1	Applied Biosystems (MT, USA)	73	Real-time PCR
Il6	16193	Mm00446190_m1	Applied Biosystems (MT, USA)	78	Real-time PCR
Prss35	244954	Mm01175001_g1	Applied Biosystems (MT, USA)	56	Real-time PCR

Genotyping PCR for KO mice

Il1f6	54448	TTGTGGATAAAAGGCTTGGAC	CTCACCCCAAGCTGTAGCAC	297	Genotyping PCR

bp: product size. **Havcr1* is the coding gene of Kim-1.

Supplemental Table 2. Potential exonic off-targets sites caused by CRISPR/Cas9 mediated gene editing.

Site name	Sequence*	Off target score	RefSeq	Locus
**Target_Ex2**	CCTAGGGTCAATCTGCAGAT**TGG**			
OT_Ex2_1	CtTgGGGTtcATCTGCAGAT**cGG**	0.8	NM_178414	Chr7:+126846768
OT_Ex2_2	tCTgGGGaCAAgCTGCAGATgaG	0.7	NM_019799	Chr7:-86743452
OT_Ex2_3	CtTtGGGaCAATCTGCAGAa**aGG**	0.7	NM_177310	Chr15:-81283393
OT_Ex2_4	CCTcGGGTCAtTCTGCAGgT**gGG**	0.6	NM_194342	Chr15:-79557471
OT_Ex2_5	CtTAaGGTCAAaCTGCtGATaaG	0.3	NM_175540	ChrX:-94530353
OT_Ex2_6	CCcAaGGTCAAgCTcCAGAT**gGG**	0.2	NM_183208	Chr14:-26475954
OT_Ex2_7	CaTAGGGcCAAcCTGgAGATTaG	0.1	NM_181547	Chr2:+68990385
OT_Ex2_8	CCaAGGGTCAcTCTaCAGAaaaG	0.1	NM_175091	Chr8:+35897617
OT_Ex2_9	CCaAGGGTCAAgCTaCAGAaaaG	0.1	NR_046475	Chr12:-110784755
OT_Ex2_10	CCcAGGGaCAATggGCAGAT**TGG**	0.1	NM_174851	Chr4:-135262004
OT_Ex2_11	CCTAGGGTgAcTgTGCAGAc**aGG**	0.1	NM_001085507	Chr2:+33263698
OT_Ex2_12	CCcAGGGTCAtTCTGggGAT**gGG**	0.1	NM_153501	Chr2:+131123152
OT_Ex2_13	CCTAGGGTtAAagTGCAGcT**gGG**	0.1	NM_008675	Chr4:+138638399
OT_Ex2_14	CCTAGGGTCAcTgTGCtGAgaaG	0.0	NR_028307	Chr8:+11694116
OT_Ex2_15	CCTgGGGTCAAcCTGCAtgT**cGG**	0.0	NM_008369	Chr14:-15181251
OT_Ex2_16	CCTAGGGcCAATCaGgAcATcaG	0.0	NM_178380	Chr8:+112078649
**Target_Ex3**	AGGGGGGATCCCACGTACAT**GGG**			
Target_Ex3_1	AGaGGGGAcCtCACtTACAT**aGG**	0.1	NM_029932	Chr11:+72343016
Target_Ex3_2	AGGGGGGtTaCCAaGTACAgGaG	0.1	NR_029414	Chr15:-63755619
Target_Ex3_3	AGGGGGcATCCaAgGTACcT**tGG**	0.0	NM_001113214	Chr2:-29749023
Target_Ex3_4	AGGGGGGcTCCCAaGTAaAg**aGG**	0.0	NM_007919	Chr4:+141375069
Target_Ex3_5	AGGGGGGATCCCcgGTgCtT**GGG**	0.0	NM_172898	Chr7:-31239146

Ex: exon. Chr: chromosome. *Mismatches from the on-target sequence are lower-case and underlined. PAM sequence is boldface.

Supplemental Table 3. Antibodies

Antibody	Source	Dilution	Antigen retrieval	Treatment
**For sections**				
Goat anti- IL-36α	R&D Systems (Minneapolis, MN, USA)	1:400	10 mM CB (pH 6.0)	105ºC, 20 min
Rabbit anti-CD3	Nichirei (Tokyo, Japan)	1:200	20 mM TB (pH 9.0)	105ºC, 20 min
Rabbit anti-Calbindin D28k	Spring Bioscience (Pleasanton, CA, USA)	1:500	10 mM CB (pH 6.0)	105ºC, 20 min
Rabbit anti-Caspase3 (active/cleaved)	Cell signaling (Danvers, MA, USA)	1:300	10 mM CB (pH 6.0)	105ºC, 20 min
Rabbit anti-Iba1	Wako (Tokyo, Japan)	1:1200	10 mM CB (pH 6.0)	105ºC, 20 min
Rabbit anti-ssDNA	Immuno-Biological Laboratories (Gunma, Japan)	1:200	-	-
Rabbit anti-αSMA	Abcam (Cambridge, UK)	1:3000	10 mM CB (pH 6.0)	105ºC, 20 min
Rat anti-B220	Cedarlane (Homby, Ontario, Canada)	1:1600	10 mM CB (pH 6.0)	105ºC, 20 min
Rat anti-BrdU	Abcam	1:200	10 mM CB (pH 6.0)	105ºC, 20 min
Rat anti-Gr1	R&D Systems (Minneapolis, MN, USA)	1:800	0.1% pepsin/0.2 N HCl	37ºC, 15 min
Rabbit anti-IL-1RL2	Abcam (Cambridge, UK)	1:100	20 mM TB (pH 9.0)	105ºC, 20 min
Rabbit anti-Prss35	Gene Tex (Irvine, CA, USA)	1:100	0.1% pepsin/0.2 N HCl	37ºC, 15 min
**For cells**				
Rabbit anti-Calbindin D28k	Spring Bioscience (Pleasanton, CA, USA)	1:100	-	-
Rabbit anti-THP	Santa Cruz Biotechnology (Dallas, TX, USA)	1:100	-	-
Rabbit anti-Hnf4α	Santa Cruz (Dallas, TX, USA)	1:100	-	-

CB: citrate buffer. TB: Tris-HCl buffer.

Supplemental Figure 1. MTEC15 cell line shows the phenotypes of PTs.

MTEC15 cell line was established from transformation-related protein 53 (Trp53)-KO mouse (accession number, CDB 0001K; Riken, Center for Developmental Biology, Kobe, Japan) [*Oncogene*, 1993, 8: 3313-22]. MTEC15 cell line was maintained in REGM BulletKit (Lonza). After stimulation with bovine serum albumin (BSA; 20 μg/mL) or transforming growth factor-β1 (TGF-β1; 5 ng/mL, R & D systems) for 24 h, epithelial morphology and gene expression was analyzed by using specific primers (Supplemental Table 1). MTEC15 cell line was incubated with labeled albumin (equivalent to 8 μg/mL BSA) by HiLyte Fluor 555 (Dojindo), fixed by 4% PFA, washed by PBS, and stained by ProLong™ Diamond Antifade Mountant with DAPI (Thermo Fisher Scientific).

MTEC15 cell line, derived from Trp53-KO mouse (accession number, CDB 0001K, Riken, Center for Developmental Biology, Kobe, Japan) [*Oncogene*, 1993, 8: 3313-22], showed epithelial cell morphology, and their size was increased by stimulation with BSA (20 μg/mL) or TGF-β1 (5 ng/mL) for 24 h (Fig. a). Furthermore, increased gene expression of PT markers, including aquaporin 1 (*Aqp1*) and low-density lipoprotein receptor-related protein 2 (*Lrp2*, also known as megalin) was detected after stimulation with BSA for 24 h (Fig. b). Albumin uptake by MTEC15 was clearly observed after 60 min of incubation (Fig. c). For immunofluorescence, a positive reaction for the PT marker, hepatocyte nuclear factor 4 alpha (Hnf4α) was observed, but expression of DT markers, including uromodulin (also known as tamm-horsfall protein THP) and calbindin D28k, was weakly detected (Fig. d).

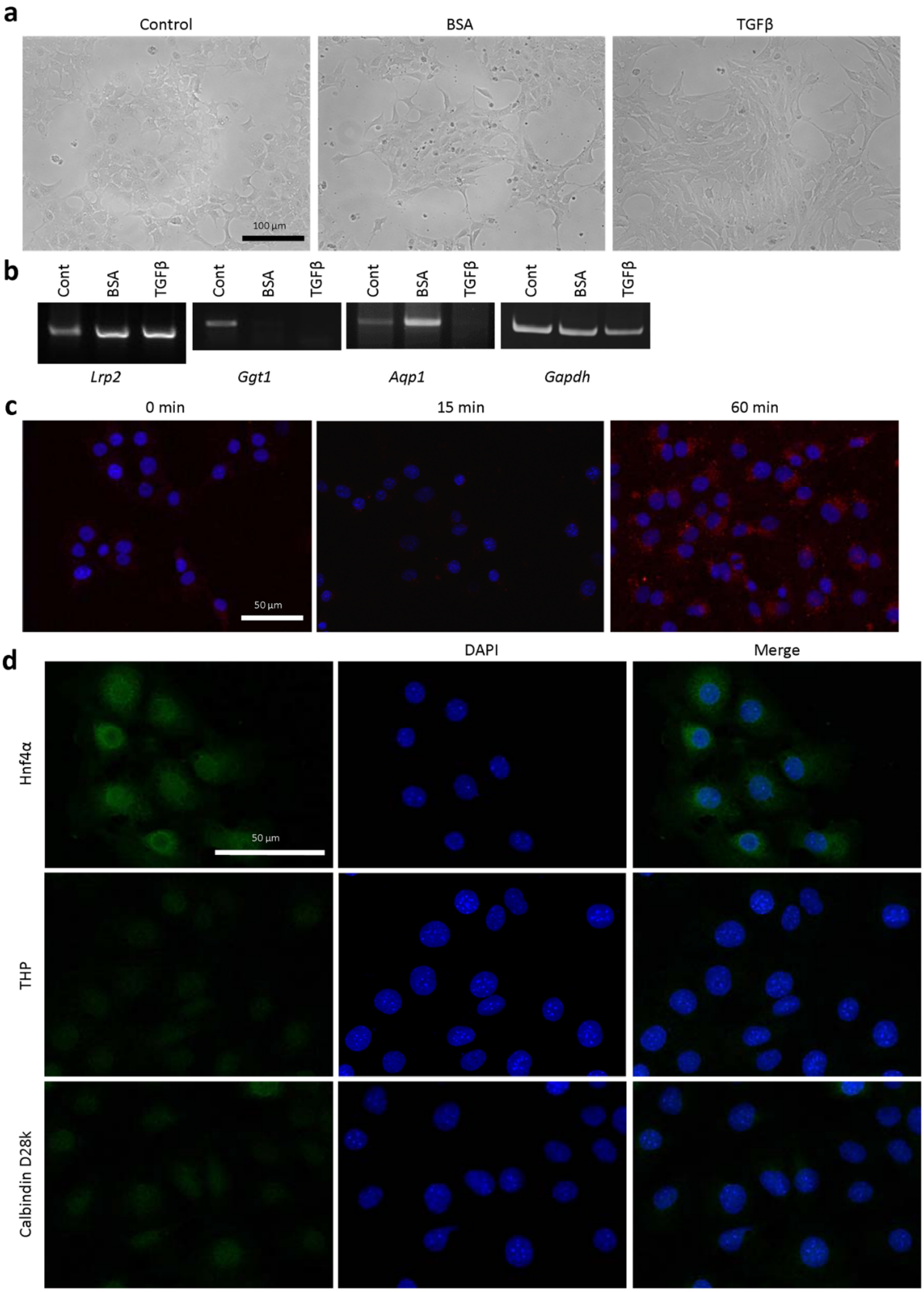

a
Control
BSA
TGFβ
100 µm
b
Cont
BSA
TGFβ
Lrp2
Ggt1
Aqp1
Gapdh
c
0 min
15 min
60 min
50 µm
d
DAPI
Merge
Hnf4α
THP
Calbindin D28k
50 µm

Supplemental Figure 2. Altered sensing-associated genes and cilia morphology in UUO kidneys.

The percentage of down- or up-regulated genes in the kidney of IL-36α-KO mice compared with that in WT mice. Microarray analysis at 48 h.

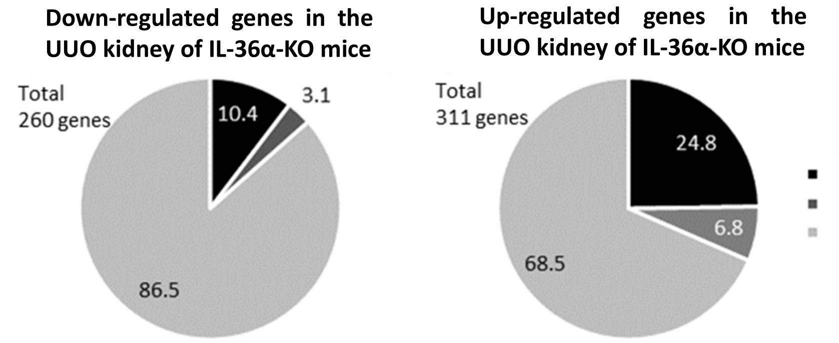

Supplement: Supplementary file 1 [file Data_Sheet_1.pdf]
